# The MicroRNA Biology of the Mammalian Nucleus

**DOI:** 10.1038/mtna.2014.40

**Published:** 2014-08-19

**Authors:** Thomas C Roberts

**Affiliations:** 1 Department of Molecular and Experimental Medicine, The Scripps Research Institute, La Jolla, California, USA; 2 Department of Physiology, Anatomy and Genetics, University of Oxford, Oxford, UK

## Abstract

MicroRNAs (miRNAs) are a class of genome-encoded small RNAs that are primarily considered to be post-transcriptional negative regulators of gene expression acting in the cytoplasm. Over a decade of research has focused on this canonical paradigm of miRNA function, with many success stories. Indeed, miRNAs have been identified that act as master regulators of a myriad of cellular processes, and many miRNAs are promising therapeutic targets or disease biomarkers. However, it is becoming increasingly apparent that the canonical view of miRNA function is incomplete. Several lines of evidence now point to additional functions for miRNAs in the nucleus of the mammalian cell. The majority of cellular miRNAs are present in both the nucleus and the cytoplasm, and certain miRNAs show specific nuclear enrichment. Additionally, some miRNAs colocalize with sub-nuclear structures such as the nucleolus and chromatin. Multiple components of the miRNA processing machinery are present in the nuclear compartment and are shuttled back and forth across the nuclear envelope. In the nucleus, miRNAs act to regulate the stability of nuclear transcripts, induce epigenetic alterations that either silence or activate transcription at specific gene promoters, and modulate cotranscriptional alternative splicing events. Nuclear miRNA-directed gene regulation constitutes a departure from the prevailing view of miRNA function and as such, warrants detailed further investigation.

## Canonical miRNA Biogenesis and Function

Microribonucleic acids (microRNAs, miRNAs, miRs) are short (~22 nucleotide), single-stranded, genome-encoded RNA molecules. miRNAs are generated by the cleavage of precursor hairpins in two sequential processing reactions. Initially, miRNAs are transcribed as long primary-miRNA (pri-miRNA) transcripts which are cleaved in the nucleus by the enzyme DROSHA (Drosha) to liberate the precursor-miRNA (pre-miRNA) hairpin.^1^ The pre-miRNA is subsequently exported from the nucleus in a process mediated by the karyopherin XPO5 (Exportin-5).^2^ Further processing by the enzyme DICER1 (Dicer) in the cytoplasm removes the loop sequence from the hairpin to produce an RNA duplex analogous to a small interfering RNA (siRNA).^3^ The double-stranded RNA duplex is then passed to an Argonaute protein (*e.g.*, AGO2, Argonaute-2) and one strand is discarded, leaving only the mature miRNA species.

The primary function of miRNAs appears to be the execution of endogenous RNA interference (RNAi) (*i.e.*, the post-transcriptional regulation of gene expression).^4^ The mature miRNA acts to guide the ribonucleoprotein complex RISC (RNA-induced silencing complex) to target mRNA transcripts at cytoplasmic Processing-bodies (P-bodies).^5,6^ Canonically, miRNAs function by binding in the 3′ untranslated region (3′ UTR) of a target mRNA, typically forming an imperfect duplex. While multiple mismatches are tolerated between a miRNA and its target, high complementarity in the miRNA “seed” region (*i.e.*, nucleotides 2–7) is generally required for effective target recognition.^7^ The degree of complementarity between the miRNA and its cognate target determines the fate of the targeted RNA. High levels of complementarity result in cleavage at a specific nucleotide position via the “slicer” activity of AGO2.^8^ Lower levels of complementarity lead to gene silencing via alternate mechanisms. Early studies showed that miRNAs induced translational repression whereby protein expression was reduced while mRNA levels were unaffected. Subsequently, other studies have shown that miRNAs can also induce mRNA decay by slicer-independent mechanisms.^9,10,11^ Importantly, there are numerous exceptions to the canonical modes of miRNA biogenesis and function.^12^

## miRNAs Are Present in the Nucleus

The prevailing view is that miRNAs function to regulate mRNA stability and translation in the cytoplasm. However, multiple studies have detected miRNAs in the nuclear compartment. For example, miR-21 was detected in both nuclear and cytoplasmic HeLa cell extracts by northern blot as early as 2004.^13^ Subsequently, Hwang and coworkers showed that miR-29b is predominantly nuclear localized in the nuclei of HeLa and 3T3 cells, whereas the related miRNA, miR-29a, is mainly located in the cytoplasm.^14^ A key difference between these two miRNAs is the presence of a hexanucleotide sequence (AGUGUU) at the 3′ terminus of miR-29b. Transplantation of this motif onto an siRNA targeted against luciferase was sufficient to bias its cellular localization towards the nucleus, thereby demonstrating that this sequence is a *bona fide* nuclear localization signal.^14^

Systematic analyses of the sub-cellular distribution of miRNAs suggest that the majority of miRNAs are present in both nuclear and cytoplasmic compartments.^15,16,17,18^ Importantly, the use of microarrays with probes that preclude the binding of pre-miRNA hairpins confirmed the presence of mature miRNA species in the nucleus (as opposed to DROSHA processing products).^16^ Small RNA sequencing of nuclear and cytoplasmic fractions from human nasopharyngeal carcinoma cells revealed substantial overlap between miRNAs found in either location (nuclear: 339 miRNAs, cytoplasmic: 324 miRNAs, 300 miRNAs overlap).^15^ Similarly, Gagnon *et al*. reported that ~75% of cellular miRNAs are present in both the nucleus and the cytoplasm of HeLa cells.^19^ Using a combination of microarray analysis and small RNA deep sequencing, Khudayberdiev *et al*. investigated nuclear miRNAs in rat primary cortical neurons.^18^ In general, each miRNA was found to be approximately two- to fourfold less abundant in the nucleus relative to the cytoplasm, although two miRNAs (miR-25 and miR-92a) showed clear nuclear enrichment. Expression levels of miRNAs in the nucleus declined during the process of post-mitotic neuronal development^18^ suggesting that nuclear miRNAs might be important for maintaining the undifferentiated state, and that the global down-regulation of nuclear miRNAs is important for cortical development.

Conflicting results have been reported regarding the localization of miR-29b. Nuclear enrichment of miR-29b was confirmed in 5-8F cells^15^ but not in HCT116 colorectal carcinoma cells^16^ suggesting that the machinery involved in the motif-dependent nuclear import of miR-29b is differentially active between cell-types. While Liao *et al*. identified a number of nuclear-enriched miRNAs in addition to miR-29b (*e.g.*, miR-1, miR-15, miR-32, and miR-148a/b), the hexanucleotide motif identified by Hwang *et al*.^14^ was not found to be over-represented among these nuclear miRNAs,^15^ indicating that distinct mechanisms are responsible for their differential sub-cellular localizations.

Some miRNAs have also been found to colocalize with specific substructures within the nucleus. A study by Politz *et al*. utilizing *in situ* hybridization in rat L6 myoblasts found that miR-206 (which is primarily expressed in skeletal muscle and regulates the process of myogenic differentiation)^20^ was expressed in the cytoplasm but was also concentrated in the granular component of the nucleolus.^21^ A follow-up miRNA profiling study showed that many mature and precursor miRNAs (mainly nonmuscle specific) localize to the nucleolus of rat myoblasts, suggesting that they may be involved in cellular housekeeping functions.^22^ Additionally, variations in the nucleolar localization of miRNAs were observed between cells in the same cultures, suggesting that sub-nuclear miRNA localization may be a transient (and perhaps regulated) process. Remarkably, the induction of cell stress by the transfection/electroporation of foreign nucleic acids, or by infection with influenza A virus, induced a shift in the sub-cellular distribution of nucleolar miRNAs to a more cytoplasmic location in HeLa cells.^23^ Taken together, these studies suggest that (i) the nucleolus is a site of storage for miRNAs which remain inactive until released by cell stress, and/or (ii) that nucleolar miRNAs function in a manner distinct from the canonical cytoplasmic RISC paradigm. Given that the nucleolus is the site of ribosomal gene transcription, ribosomal RNA maturation and RNA editing, it is possible that nuclear miRNAs participate in the regulation of these processes, or are themselves subject to RNA editing.^24,25,26^

## miRNA Pathway Components Shuttle Between Cytoplasm and Nucleus

Multiple studies have identified components of the miRNA processing pathway in the nucleus. All four human Argonaute proteins and other RNAi factors (*i.e.*, DICER1, TARBP2 and TNRC6A (GW182)) have been detected by western blot in nuclear lysates from a variety of human cell lines.^19,27,28,29^ Nuclear extracts retain the catalytic activities of AGO2 and DICER1 *in vitro*^19,27^ suggesting that active RISC complexes are present in the nucleus. There have been conflicting reports regarding the composition of the nuclear RISC complex. Using fluorescence correlation spectroscopy, Ohrt *et al*. showed that two distinct forms of RISC exist in the nuclear and cytoplasmic compartments (nRISC and cRISC respectively).^30^ The size of these complexes was markedly different. cRISC was determined to be ~3 MDa consistent with previous estimates,^31^ whereas nRISC was much smaller at ~150 kDa (which is close to the size of AGO2 alone). Asymmetric siRNA strand incorporation was found to be similar between cRISC and nRISC suggesting a common loading mechanism.^30^

In contrast, several studies have reported that Argonaute proteins form multi-protein complexes in the nucleus. The RNAi factors AGO2, TNRC6A, DICER1, and TARBP2 were shown to be directly associated by pairwise coimmunoprecipitation in HeLa cell nuclei.^19^ All four proteins were detected in a high molecular weight fraction following size exclusion chromatographic separation of nuclear lysates, suggesting that the nuclear RNAi machinery forms an intact protein complex.^19^ Similarly, TNRC6A and AGO2 were found to colocalize with miRNAs, and form punctate foci in the nucleus.^32^ In further support, it was shown that both AGO1 and AGO2 are present in the chromatin fraction of HeLa nuclear extracts, and coprecipitate with multiple RNAi factors (TNRC6A, TNRC6B, hnRNPs, RNA helicases) and epigenetic modifier proteins (CBX3, TIF1B, SUV39H1, EHMT2).^33^

The translocation of endogenous miRNAs from cytoplasm to nucleus has been visualized directly by Földes-Papp *et al*. who utilized optoporation to selectively permeabilize single cells to superquencher molecular probes complementary to the mature form of miR-122.^34^ miR-122 probes were initially localized to the cytoplasm, but fluorescence signal was also detected in the nucleus ~5 minutes post-optoporation, thereby demonstrating nuclear import.^34^ Furthermore, the delivery of siRNAs directly to the cytoplasm by microinjection led to efficient silencing of the nuclear-retained RN7SK transcript, suggesting that RISC is loaded in the cytoplasm and subsequently shuttled to the nucleus.^30^ In further support, multiple RISC loading factors (*i.e.*, HSP90AA1, TSN, TSNAX, AHA1, FKBP4, CDC37, and PTGES3) are restricted to the cytoplasm, and *in vitro* loading of RISC with radiolabeled duplex RNAs is impaired in nuclear extracts.^19^ Together these observations strongly suggest that RISC loading occurs exclusively in the cytoplasm prior to nuclear import. The spatial separation of miRNA processing and RISC loading between nucleus and cytoplasm permits tighter control of flux through the miRNA processing pathway, thereby avoiding toxicity associated with pathway oversaturation.^35^

The observations that (i) mature miRNAs are present in the nucleus, and (ii) nuclear RISC is deficient with respect to miRNA loading, necessitate the existence of cellular machinery for shuttling RISC across the nuclear envelope. Such mechanisms have been identified in *C. elegans* through genetic screens,^36^ and in *Tetrahymena*,^37^ but were unknown in mammalian cells until recently. The translocation of molecules through the Nuclear Pore Complex (NPC) is mediated by a family of proteins called the karyopherins (for example, the nuclear export of pre-miRNA hairpins by the karyopherin XPO5, as mentioned above).^38^ Several studies have identified karyopherins that are involved in the transport of RISC between the nucleus and the cytoplasm in mammals. For example, Castanotto *et al*. showed that XPO1 (Exportin-1) facilitates the transport of mature miRNAs from the cytoplasm to the nucleus in human cells.^39^ Inhibition of XPO1 with Leptomycin B prevented the accumulation of miRNAs in the nucleus, and synthetic dicer substrate siRNAs competed with endogenous miRNAs for nuclear import. Furthermore, coimmunoprecipitation studies showed that XPO1 is associated with the RISC components (AGO1, AGO2, DICER1, RHA, TNRC6B) and the transcriptional regulators (EZH2, TOP2A, and MTA) suggesting that XPO1 may facilitate the import of intact protein complexes loaded with miRNAs.^39,40^ Interestingly, the P-body-associated RNAi factor, TNRC6A, acts as a navigator protein that facilitates the shuttling of AGO2 to and from the nucleus in conjunction with XPO1.^32^ The TNRC6A protein contains both nuclear import and export signals and was shown to mediate nRISC export in an XPO1-dependent manner, suggesting that this karyopherin regulates both nuclear import and export of miRNAs.^32^

The karyopherin IPO8 (Importin-8) has also been implicated in the nuclear import of miRNAs. In HeLa cells, IPO8 was found to associate with AGO2 in the nucleus and in P-bodies in the cytoplasm.^41^ siRNA-mediated knockdown of IPO8 resulted in a reduction of AGO2 in the nucleus while the total cellular concentration of AGO2 was unchanged, indicating a shift in the sub-cellular distribution of AGO2. Importantly, some residual AGO2 protein remained in the nucleus after IPO8 knockdown consistent with multiple redundant cellular pathways for Argonaute-nucleocytoplasmic shuttling.^41^

**Figure 1:**
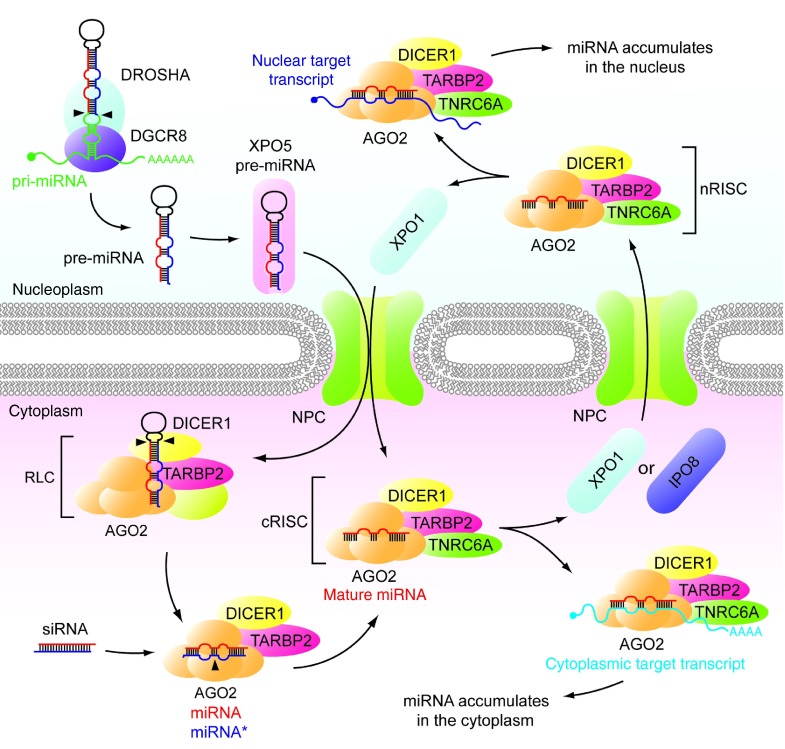
**Nucleocytoplasmic shuttling of miRNAs. **miRNAs are initially transcribed as long pri-miRNA transcripts in the nucleus. A complex of DGCR8 and DROSHA recognizes hairpin structures located on the pri-miRNA. DROSHA liberates the pre-miRNA by cleaving at the base of the hairpin (indicated by black arrowheads). Export of the pre-miRNA through the Nuclear Pore Complex (NPC) is mediated by the karyopherin XPO5. In the cytoplasm, the pre-miRNA is further processed by the RISC loading complex (RLC) which consists of an Argonaute protein (AGO2 is depicted here), DICER1, TARBP2, and other factors (which include the cytoplasm-restricted HSP90AA1, TSN, TSNAX, AHA1, FKBP4, CDC37, and PTGES3). The loop sequence of the hairpin is removed by DICER1 cleavage (indicated by black arrowheads). Subsequently, TARBP2 facilitates the loading of the RNA duplex into AGO2. The mature form of the miRNA is shown in red whereas the passenger strand (miRNA*) is shown in blue. Exogenous RNA duplexes (*i.e.*, siRNAs) can also enter AGO2 at this stage in the processing pathway. One of the two strands is retained in AGO2 and the other degraded. The loaded cytoplasmic RISC (cRISC) complex, which contains the mature miRNA species and the silencing factor TNRC6A, is now capable of binding to cytoplasmic target transcripts. In the case of high miRNA-target complementarity AGO2 cleaves at the point indicated by the black arrowhead. Alternatively, cRISC can be imported into the nucleus by XPO1 or IPO8. TRNC6A acts as a navigator protein during this process. Nuclear RISC (nRISC) maintains a similar composition to cRISC although or may also exist as AGO2-miRNA complex alone (not depicted). The nRISC complex may bind additional nuclear factors or the Argonaute protein may form a distinct multi-protein complex (*e.g.*, RITS, not depicted). nRISC binds to complementary nuclear transcripts or, in the absence of nuclear targets, be exported to the cytoplasm in a process facilitate by XPO1. Differential accumulation of miRNAs in the cytoplasm or nucleus is, in part, determined by the location of target transcripts.

Several lines of evidence suggest that sub-cellular miRNA localization may be determined by the location of their target transcript(s). For example, Berezhna *et al*. showed that an siRNA targeting RN7SK was predominantly localized to the nucleus, whereas an siRNA targeting the cytoplasmic hepatitis C virus replicon RNA was retained in the cytoplasm.^42^ Separately, Ahlenstiel *et al*. showed that complexes of AGO1 and an siRNA targeting the 5′ LTR region of the integrated HIV provirus were localized to the nucleus of HIV-infected cells but not in HIV-naïve cells.^43^ Intriguingly, these studies suggest that siRNAs/miRNAs become “trapped” and accumulate in the specific sub-cellular compartment in which their cognate targets reside. Nuclear accumulation of Argonaute-bound small RNAs is also dependent on the duration of interaction with the target transcript as a RN7SK-targeted siRNA with central mismatches (which inhibit the slicer activity of AGO2 and more closely mimic endogenous miRNA structure) showed increased nuclear AGO-siRNA accumulation relative the equivalent siRNA with no mismatches.^30^ These data suggest that all (or most) miRNAs and exogenous siRNAs are nonspecifically shuttled between the nucleus and the cytoplasm, such that the pool of AGO-miRNA complexes can scan each compartment for target transcripts (**Figure 1**). The observation that the majority of mature miRNAs that are present in the cytoplasm can also be detected in the nucleus lends credence to this idea.^15,19^

## Nuclear miRNA Function

**Figure 2:**
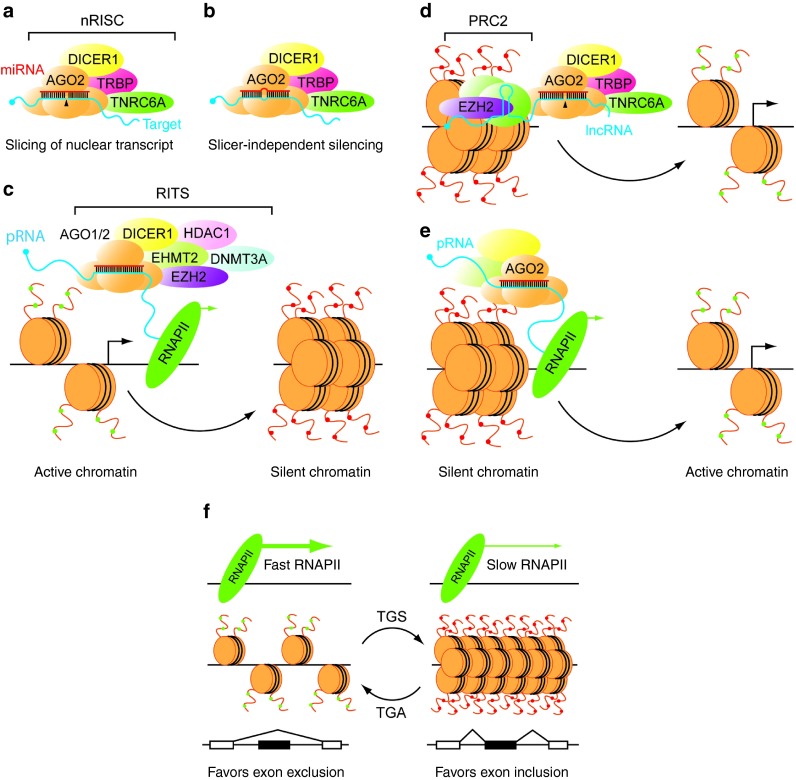
**Gene regulatory mechanisms of nuclear miRNAs.** miRNAs can induce Post-Transcriptional Gene Silencing (PTGS) of a target transcript via the nuclear RNA Induced Silencing Complex (nRISC). (**a**) Silencing can occur via AGO2-mediated target slicing leading to transcript degradation (indicated by black arrowhead), or (**b**) by a slicer-independent mechanism. (**c**) Transcriptional Gene Silencing (TGS) occurs when a miRNA directs the RNA-Induced Transcriptional Silencing complex (RITS) to low-copy promoter RNA (pRNA) transcripts. RITS consists of chromatin remodeling activities (HDAC1, EHMT2, and EZH2) in addition to the DNA-methyltransferase, DNMT3A which facilitate the transition from a transcriptionally active chromatin structure to silent heterochromatin. Several putative mechanisms of Transcriptional Gene Activation (TGA) have been proposed. (**d**) At certain loci, lncRNAs silence gene expression by recruiting transcriptional repressors (*e.g.*, Polycomb Repressive Complex 2, PRC2). miRNA-mediated silencing of the lncRNA disrupts the recruitment of silencing factors leading to activation of the target loci. (**e**) Alternatively, miRNAs may induce TGA by recruiting a protein complex containing transcriptional activators, as cleavage of the pRNA is not necessarily required for activation to occur. (**f**) miRNAs can influence alternate splicing decisions at specific exons. miRNA-mediated modulating of the chromatin landscape at the targeted exon effects the rate of RNAPII procession. Faster RNAPII procession through open chromatin promotes exon exclusion whereas slower RNAPII procession through compacted chromatin favors exon inclusion.

The functions of nuclear miRNAs are currently not well understood. The nuclear localization of mature miRNA species and RNAi factors, in addition to the existence of mechanisms for nucleocytoplasmic RISC shuttling, strongly suggest that post-transcriptional gene silencing occurs in the nucleus (**Figure 2a**,**b**). Early studies in *C. elegans* showed that dsRNAs complementary to intronic regions were capable of pre-mRNA silencing (if the pre-mRNA transcript was sufficiently long-lived)^44^ thus demonstrating that nuclear transcripts are susceptible to RNAi. Similar results were later observed in human (HeLa) cells whereby siRNAs targeted against the RN7SK and U6 small nuclear RNA transcripts induced potent post-transcriptional silencing.^27,30^ Other nuclear-retained transcripts are also susceptible to RNAi-mediated degradation such as the toxic mutant DMPK transcript which is the cause of myotonic dystrophy,^45^ and the highly abundant long noncoding RNA (lncRNA) MALAT1.^19,32^

The notion that miRNAs have general functional roles in the nucleus is further supported by unbiased analysis of miRNA-mRNA-Argonaute interactions in mouse brain using high-throughput sequencing of RNAs isolated by crosslinking immunoprecipitation (HITS-CLIP).^46^ While the majority (40%) of AGO-mRNA tags mapped to the 3′ UTR regions of mRNAs (consistent with the canonical view of miRNA function) a substantial number (12%) mapped to intronic sequences (which are nuclear-retained) indicative of Argonaute activity in the nucleus. Furthermore, 4% of AGO-mRNA tags mapped to lncRNAs, suggesting that miRNAs contribute to the regulation of the noncoding RNA transcriptome. Indeed, immunoprecipitation of Argonaute followed by microarray analysis identified the lncRNAs MALAT1 and H19 as some of the most highly enriched target transcripts.^41^ Subsequently, direct miRNA targeting of nuclear-retained noncoding transcripts has been demonstrated in specific cases (*i.e.*, miR-9, miR-210, and miR-671 regulate MALAT1, XIST, and CDR1 respectively).^47,48,49^ Additionally, a nuclear localized miRNA, miR-709, acts as a post-transcriptional regulator of the primary-miR-15a/miR-16-1 transcript indicating that nuclear miRNAs can regulate the biogenesis of other miRNAs in a hierarchical manner.^50^ Together these studies provide compelling evidence for miRNA-mediated regulation of nuclear transcript stability.

## miRNAs in Epigenetic Regulation

Small RNA-mediated transcriptional gene silencing (TGS) is uncontroversial in fission yeast, *Drosophila* and *Arabidopsis*,^51,52,53,54^ but has only been known to occur in human cells since 2004.^55^ Exogenous small RNAs guide the RNA Induced Transcriptional Silencing (RITS) complex to promoter-associated transcripts which triggers alterations in chromatin structure and promoter CpG methylation leading to transcriptional gene silencing (*i.e.*, epigenetic-TGS).^55,56^ In other cases, siRNAs complementary to transcription start sites inhibit assembly of the pre-initiation complex and therefore sterically inhibit RNA polymerase II binding and procession.^57,58^

The observation that synthetic small RNAs could directly induce gene-specific changes in transcriptional activity led to the pursuit of endogenous miRNAs which might exhibit similar effects. By searching for completely complementary miRNA target sites in promoter regions, Kim *et al*. identified the *POLR3D* promoter as a target of miR-320.^59^ Transfection of HEK293 cells with miR-320 mimics induced enrichment of AGO1 and EZH2 at the *POLR3D* locus, leading to heterochromatinization and transcriptional gene silencing^59^ (**Figure 2c**). Genome-wide analysis of potential miRNA targets revealed that human promoters are enriched for miRNA seed matches, suggesting that miRNA-mediated TGS is likely to be a general phenomenon.^60^ Multiple other examples of miRNA-mediated epigenetic silencing have since been reported at specific promoters,^61,62,63^ and in the regulation of cellular senescence,^64^ granulopoiesis^65^ and nerve regeneration^66^ via the targeting of multiple genes.

Paradoxically, small RNAs (including miRNAs) can also induce transcriptional gene activation (TGA).^29,67,68,69^ Several common features suggest that the TGA and epigenetic-TGS are functionally related^70^ as both processes are mediated by Argonaute proteins,^29,62,71^ and are dependent upon the presence of promoter proximal noncoding transcripts.^69,72,73,74^ However, the mechanism of miRNA-mediated TGA is currently poorly understood. One possibility is that miRNAs silence promoter proximal lncRNA transcripts which are themselves *cis* negative regulators of their adjacent genes. As such, the promoter-targeting miRNA acts to “repress a repressor” leading to transcriptional activation of the neighboring gene^69,75^ (**Figure 2d**). Alternatively, AGO-miRNA complexes may recruit positive epigenetic regulators to target promoters. This recruitment hypothesis is supported by the observation that miRNA-mediated transcriptional activation was shown to be independent of target RNA cleavage^76^ (**Figure 2e**).

In the first reported case of miRNA-mediated TGA, miR-373 was found to activate transcription of *CDH1* and *CSDC2* (both of which contain highly complementary target sites in their respective promoters).^77^ Subsequently, it was shown that miR-205 directs transcriptional activation of the interleukin genes *IL24* and *IL32* though promoter interactions.^78^ However, in these cases the complementarity between miR-205 and the promoter target sites was limited outside of the seed region, thereby indicating that high levels of complementarity are not required for miRNA-induced TGA, and that miRNA target sites in promoters are likely to be widespread. In the most comprehensively studied example to date, miR-589 was shown to activate transcription of the inflammatory regulator *PTGS2* (COX-2) by targeting two primate-conserved target sites on a sense promoter RNA.^76^ Activation of *PTGS2* required recruitment of AGO2 and TRNC6A to the *PTGS2* promoter RNA, and was coincident with promoter enrichment of active histone post-translational modifications. A highly interesting further finding was that miR-589 also activated expression of a distal pro-inflammatory gene, *PLA2G4A*, the promoter of which was found to be in close contact with the *PTGS2* promoter by chromosome conformation capture (3C) analysis.^76^ This observation is consistent with the idea that promoter proximal lncRNA transcripts act as chromatin signatures (in three-dimensional space) that permit small RNA guided ribonucleoprotein complexes to target specific genomic loci.

## miRNAs in the Regulation of Alternative Splicing

Building on small-RNA-directed TGS studies, Alló *et al*. showed that targeting an intronic region of the FN1 pre-mRNA with an siRNA resulted in local chromatin remodeling and altered splicing of the adjacent exon.^79^ The authors proposed that chromatin compaction at specific intron/exon loci leads to slowing of RNA polymerase II procession which favors exon inclusion (consistent with the kinetic model of coupling between transcription and alternative pre-mRNA splicing)^80^ (**Figure 2f**). Knockdown and overexpression of AGO1, AGO2, and DICER1 influenced splicing decisions at a number of alternatively spliced exons,^33,79^ suggesting a general role for the RNAi machinery in the regulation of splicing. Furthermore, analysis of chromatin-bound Argonaute proteins by coimmunoprecipitation and mass spectrometry identified multiple AGO-associated splicing factors (U2 and U5 snRNP core subunits, SRSF1, SRSF3, SRSF7, SRSF10, PTBP1, PTBP2, KHDRBS1, and snRNAs)^33^ demonstrating a direct link between splicing factors and nuclear RISC.

Independently, data from the Corey lab suggested that nuclear small RNAs can induce alternative splicing events via a different mechanism.^81^ siRNAs targeting the pre-mRNAs of *SMN2* and *DMD* (genes involved in the pathogenesis of spinal muscular atrophy and Duchenne muscular dystrophy respectively) were capable of inducing either exon inclusion or exon skipping at therapeutically relevant exons. In contrast with Alló *et al*., these siRNA-directed alternative splicing events were not dependent on alterations in chromatin structure. Inhibition of AGO2 (but not AGO1) diminished these effects on splicing and AGO2 was specifically recruited to the target transcript in siRNA-transfected cells. Interestingly, target cleavage by the slicer activity of AGO2 was not detected (even with completely complementary effector duplexes) suggesting a novel cleavage-independent role for AGO2 in the nucleus.

Given the success of steric block antisense oligonucleotides for modulating alternative splicing^82^ it is possible that AGO-miRNA complexes might function in an analogous manner. The formation of a ternary complex between the target transcript and a loaded-RISC might conceal splicing recognition motifs, thereby precluding binding of splicing factors and modulating pre-mRNA splicing events. Such a steric block functionality for AGO-miRNA complexes has been previously identified in the case of miR-122, which binds to sites at the 5′ terminus of the hepatitis C viral RNA, thereby physically shielding it from exonucleolytic degradation.^83^ Taken together, these findings strongly support the hypothesis that RNAi factors contribute to the regulation of alternative splicing via both epigenetic and nonepigenetic mechanisms.

## Perspectives

In conclusion, mature miRNAs, and their associated RNAi factors, are shuttled between the cytoplasm and the nucleus. When in the nucleus, miRNAs can influence gene expression via a variety of mechanisms including post-transcriptional silencing, transcriptional gene silencing, gene activation and modulation of alternative splicing. However, many mechanistic details remain unclear and the full extent of nuclear miRNA function is currently unknown. An intriguing possibility, supported by a growing body of evidence, is that miRNAs can act as regulators of the epigenome through interactions with lncRNAs which are increasingly being recognized as epigenetic regulators that determine cell fate and can facilitate information transfer between the external environment and the genome.^84^ Additionally, the RNAi machinery has recently been shown to be involved in directing the DNA damage response following double-strand breaks^85^ and, in *Drosophila*, AGO2 has been shown to influence chromatin structure by regulating CTCF binding^86^ (although such a function has yet to be identified in mammals). These observations point to the widespread involvement of miRNAs and the RNAi machinery in nuclear processes beyond the control of transcription and alternative splicing.

It should also be noted that a plethora of other small RNA species are present in the nucleus including transcription initiation RNAs (tiRNAs),^87^ endo-siRNAs,^88,89^ and PIWI-interacting RNAs (piRNAs).^90^ All of these small RNAs have been implicated in transcriptional and post-transcriptional gene regulatory events but do not fit the precise criteria required to be considered miRNAs (*i.e.*, they are not ~22 nucleotides in length and/or are not derived from hairpin precursors).

The role of miRNAs in the nucleus has so far been largely ignored. This is likely due, in part, to the dominance of canonical ideas of miRNA function. Very few miRNA profiling studies have compared the nuclear and cytoplasmic compartments with the vast majority of studies assuming that miRNA levels in total cellular RNA are the only biologically relevant metric. It is also highly probable that the majority of miRNAs that function in the nucleus simultaneously regulate transcript stability in the cytoplasm, meaning that differentiating putative nuclear functions from well-established cytoplasmic activities is nontrivial. Furthermore, a plethora of research tools are available that are focused towards, and have greatly facilitated conventional miRNA research but which are not necessarily applicable to the study of noncanonical miRNA functions. These include miRNA sponges, miRNA sensors, and miRNA target prediction algorithms (the majority of which only search for matches in 3′ UTRs of protein-coding genes)^7,91^ (Note: Some tools for searching any user-defined sequence for miRNA targets (RegRNA)^92^ and databases of miRNA-lncRNA interactions (DIANA-LncBase)^93^ are available to facilitate future investigations of nuclear miRNAs). Claims surrounding nuclear miRNAs also face skepticism due to concerns about the purity of nuclei preparations. However, some researchers have taken steps to demonstrate the absence of contaminating factors (*e.g.*, Calnexin derived from the membrane of the endoplasmic reticulum),^19^ and corroborating evidence from microscopy studies and the silencing of nuclear-retained transcripts are less easily dismissed as experimental artefacts. Lastly, many of the proposed nuclear functions of miRNAs are experimentally difficult to demonstrate relative to cytoplasmic post-transcriptional gene silencing which can be established easily with conventional gene expression assays.

While the majority of reports have focused on the canonical mode of miRNA action, the studies presented here emphasize the need to broaden the search when investigating miRNA gene function. As interest in nuclear miRNAs grows it is likely that novel therapeutic targets will emerge. In this instance, a nuclear miRNA which participates in a pathological gene regulatory event might be inhibited using a conventional anti-miRNA oligonucleotide strategy.^94^ Conversely, multiple studies have demonstrated small RNA-mediated transcriptional modulation using exogenous oligonucleotides. This mode of gene regulation could be used to manipulate the expression of disease-related genes. Indeed, both TGS and TGA show promise as therapeutic strategies *in vivo*.^75,95,96^ As a result, the functions of miRNAs in the mammalian nucleus present exciting possibilities for novel therapeutic intervention.
